# Antibiotic cement-coated plate is a viable and efficient technique for the definitive management of metaphyseal septic nonunions of the femur and tibia

**DOI:** 10.1590/0100-6991e-20223060-en

**Published:** 2022-12-02

**Authors:** FERNANDO BIDOLEGUI, MARIANO CODESIDO, SEBASTIÁN PEREIRA, AGUSTÍN ABRAHAM, ROBINSON ESTEVES PIRES, VINCENZO GIORDANO

**Affiliations:** 1 - Hospital Sirio Libanés, Servicio de Ortopedia y Traumatología - Buenos Aires - Argentina; 2 - Hospital Universitario Austral, Servicio de Ortopedia y Traumatología - Buenos Aires - Argentina; 3 - Universidade Federal de Minas Gerais, Departamento do Aparelho Locomotor - Belo Horizonte - MG - Brasil; 4 - Hospital Municipal Miguel Couto, Serviço de Ortopedia e Traumatologia Prof. Nova Monteiro - Rio de Janeiro - RJ - Brasil; 5 - Clínica São Vicente, Rede D’or São Luiz, Ortopedia - Rio de Janeiro - RJ - Brasil

**Keywords:** Bone Diseases, Wound Infection, Infection Control, Polymethyl Methacrylate, Pseudoartrose, Osteomielite, Infecção dos Ferimentos, Polimetil Metacrilato

## Abstract

**Objective:**

the management of septic metaphyseal nonunions is challenging, with inconsistent outcomes. Antibiotic cement-coated implants have been demonstrated good outcome for diaphyseal infected nonunions, however there is no data in metaphyseal infected nonunions.

**Methods:**

fifteen adult patients with septic metaphyseal nonunions of the femur or tibia were treated with antibiotic cement-coated plates. The antibiotic cement-coated plate was prepared with either gentamicin or vancomycin. Outcome measures were infection control, bone healing, return to pre-injury level on daily activities, and quality of life at the last follow-up visit. A p value of <5% was considered significant.

**Results:**

Methicillin-susceptible S. aureus was isolated in 53.3% cases. Average postoperative follow-up time was 18 months. Local infection control and radiographic bone healing were adequately achieved in 93.3% patients. No patient presented recurrent symptoms of surgical site infection. Fourteen patients reported to be either able, or on the same level as before injury, with 73.3% reporting no problems in all five dimensions of the EQ-5D-3L. Persistent infection was the only variable associated with a reduced long-term quality of life.

**Conclusion:**

antibiotic cement-coated plate is a viable and efficient surgical technique for the definitive management of juxta-articular metaphyseal septic nonunions of the femur and tibia.

## RESUMO

**Introdução:**

implantes revestidos de cimento com antibiótico vêm demonstrando bons resultados no tratamento da pseudoartrose infectada da diáfise, no entanto seu uso na metáfise dos ossos longos ainda é pouco explorado. Neste estudo relatamos uma série de casos de pseudoartrose infectada da metáfise do fêmur e da tíbia tratados com o uso de placas revestidas de cimento com antibiótico.

**Métodos:**

Os antibióticos usados foram gentamicina e/ou vancomicina. Os desfechos analisados na última visita ambulatorial foram controle de infecção, consolidação óssea, retorno às atividades diárias e qualidade de vida. Regressão linear bivariada foi usada para avaliar fatores individuais que afetaram a qualidade de vida dos pacientes. Um valor p<5% foi considerado estatisticamente significativo.

**Resultados:**

quinze pacientes adultos foram incluídos no estudo. S. aureus suscetível à meticilina foi isolado em 53,3% dos casos. O tempo médio de acompanhamento pós-operatório foi de 18 meses. Controle local da infecção e consolidação óssea radiográfica foram alcançados em 93,3% dos pacientes. Nenhum paciente apresentou sintomas recorrentes de infecção de sítio cirúrgico. Quatorze pacientes relataram ser capazes, mas não no nível pré-lesional ou no mesmo nível de antes da lesão, com 73,3% relatando nenhum problema em todas as cinco dimensões do EQ-5D-3L. Infecção persistente foi a única variável associada à redução da qualidade de vida a longo prazo.

**Conclusão:**

A placa revestida de cimento com antibiótico mostrou-se uma técnica cirúrgica viável e eficiente para o tratamento da pseudoartrose infectada da metáfise do fêmur e da tíbia.

## INTRODUCTION

The treatment of infected metaphyseal pseudarthrosis represents a challenging situation for patients, their families, and the orthopedic trauma team, with inconsistent results^1^. Several causes can contribute to nonunion, including the patient’s immunological status, the severity of the injury, and the degree of vascular damage during fracture reduction and fixation^2^. The presence of infection has also been considered a potential cause of nonunion, making treatment even more difficult^2^ ^,^ ^3^. Recognizing the pathogen and optimizing the patient’s biology and stability at the nonunion site are essential for a successful outcome^4^. Therefore, both bone healing and adequate management of the infection require a multimodal and multidisciplinary approach, consisting of the identification and management of the host’s systemic and local risk factors, sequential debridement of the infected wound, adequate bone stability, soft tissue coverage, and use of systemic and/or local antibiotics^5^ ^,^ ^6^.

In current clinical practice, staged surgical treatment has increasingly been considered in the management of infection after osteosynthesis (IAO), taking into account the time of onset of infection and implant stability, among some other critical aspects in decision making^5^. Recent evidence suggests that highly virulent and/or multidrug-resistant organisms and a mature biofilm can negatively impact the final outcome^5^. Furthermore, although a more conservative approach with implant retention can be used for early, and some late, cases of IAO, it is imperative to remove and replace the implant in the setting of an infected metaphyseal pseudarthrosis^2^ ^,^ ^5^ ^,^ ^6^.

Currently, both the induced membrane technique and the local use of antibiotics at the infection site through different carrier vehicles have gained increasing attention^7^ ^,^ ^8^. For adequate antibacterial efficacy, the local concentration of the antibiotic must exceed the minimum inhibitory concentrations (MIC) of infectious pathogens^9^. Until now, polymethylmethacrylate (PMMA) has been the most used carrier vehicle for the local administration of antibiotics, assuming that this drug will be gradually released to deliver higher local concentrations, being able to exceed the required MIC^8^ ^,^ ^10^ ^-^ ^13^. Antibiotic PMMA carriers can be used as small beads or cord-shaped granules, structured cylinders for mechanical stabilization of a segmental defect, or coating of osteosynthesis material such as an intramedullary nail (IMN) or plate. Although the treatment of infected long bone diaphysis pseudarthrosis using intramedullary implants coated with antibiotic cement has shown good results, in some clinical situations, such as in the case of infected pseudarthrosis of the metaphyseal region, in which one of the bone segments is very short, bone stability is best achieved by using an extramedullary internal implant^14^. We report a case series with 15 patients diagnosed with infected metaphyseal pseudarthrosis of the femur or tibia, treated with debridement of the infection focus and fixation with an antibiotic cement-coated plate.

## METHODS

### Research subjects and preoperative evaluation

This is a retrospective cohort study carried out in three level-I trauma centers, two university hospitals in Argentina and one regional hospital in Brazil. We included all adult patients with infected pseudarthrosis of the metaphysis of the femur or tibia treated with antibiotic cement-coated plates between 2014 and 2019, with a minimum follow-up of eight months. Pseudarthrosis was defined as failure of union nine months after the initial injury, with no signs of bone union progression on serial radiographs taken over a period of three consecutive months, or absence of union in case of implant failure^1^. Patients who did not complete the minimum follow-up time and those who had incomplete medical records were excluded from the study. The study was approved by the Ethics in Research Committees of the institutions (N° 4 03-2021) and informed consent forms were obtained from all subjects.

The preoperative evaluation included patient-specific information, past pathological history, physical examination, and laboratory and imaging studies. We recorded the number of previous surgeries and the type of implant used in the index surgery, and studied acute phase inflammatory markers, such as erythrocyte sedimentation rate (ESR) and C-reactive protein (CRP), and nutritional markers. We evaluated the type of pseudarthrosis (hypertrophic, oligotrophic or atrophic), the bone stock at the nonunion site, and misalignment of the axis of the affected lower limb with radiographs and computed tomography. In addition, all cases were biopsied before revision surgery to identify the pathogen.

### Surgical procedure and hospitalization course

**Figure 1:**
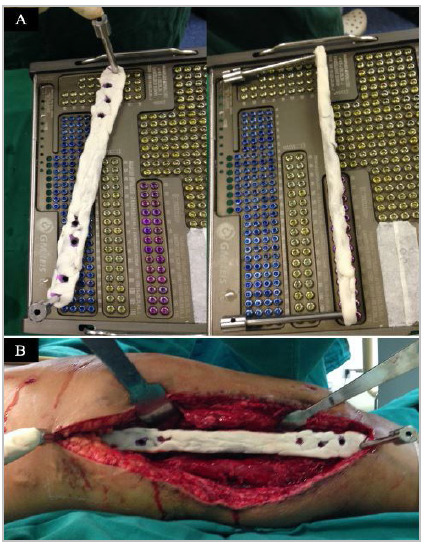
A. Intraoperative photographs of the distal periarticular locking plate of the femur covered with antibiotic-impregnated bone cement. Note that the locking turret is held in place of the holes to be used; B. Intraoperative image of the approach, showing the antibiotic cement-coated plate.

Patients were operated on without administration of antibiotics before revision surgery. After removal of the failed implant, a complete debridement of the pseudarthrosis area was performed and at least three bone tissue samples were collected for culture and histopathology. Intraoperative antibiotics were administered only after obtaining surgical cultures. Revision surgery was performed according to the principles of the ‘Diamond concept’^1^ ^,^ ^15^. The antibiotic cement coated plate was prepared on a sterile auxiliary table with 4g of gentamicin or vancomycin for each 40g of bone cement, depending on the previous identification of the pathogen^16^ ^,^ ^17^. A pre-shaped anatomical locked plate was used in all cases. Before covering the plate, the locking sheaths were placed covering the locking holes that would be used so that the cement would not interfere with the locking mechanism^14^. A 2.0 to 3.0-mm layer of cement was applied to both surfaces of the implant and, before complete polymerization of the cement, the locking sheaths were removed and the locking holes were completely cleared (Figure 1). When a bone defect was observed after debridement, we used the induced membrane technique^7^, taking care to cover the bone ends proximally and distally. Furthermore, in cases where we detected malalignment of the axis of the operated segment, it was aligned based on the assessment of the anatomical and mechanical axes of the contralateral lower limb. Finally, the soft tissue envelope was inspected and reconstructed with local flaps, when necessary.

Postoperatively, patients received broad-spectrum intravenous (IV) antibiotics until results of culture and susceptibility testing were available, at which point therapy was changed to IV antibiotics targeted at the specific pathogen(s). Intravenous antibiotic therapy was administered in a hospital setting for a minimum of three weeks, followed by an oral regimen for up to 12 weeks. Pharmacological thromboprophylaxis with 40mg of subcutaneous enoxaparin was administered for three weeks and mechanical thromboprophylaxis was encouraged from the first postoperative day, with active and passive joint mobilization, muscle contraction, and weight bearing as tolerated, with the use of two crutches or a walker. Nutritional support, including oral nutritional supplements, and guidance were given to malnourished patients and those who reported not eating.

### Evaluation of results

After discharge, patients were followed up in outpatient visits at three, six, and 12 weeks, six and 12 months, and once a semester after the first year. Outcomes assessed were infection control, bone healing, return to pre-injury level in daily activities, and quality of life at the last follow-up visit. During follow-up visits, clinical (presence of local pain, inability to support body weight, recurrent drainage, and local heat, redness, and swelling) and laboratory (ESR and CRP) markers were evaluated to exclude recurrent or persistent infection. The infectious condition was considered controlled when the acute phase inflammatory proteins showed a reduction according to the reference values (ESR 30mm/1^st^ hour and CRP 10mg/dl). Presence of bone continuity in three cortices observed in two orthogonal views or the complete disappearance of the anterior fracture line was defined as radiographic bone consolidation^18^ ^,^ ^19^. Return to pre-injury, daily activities level was assessed according to the criteria of Peek et al.^20^ modified into ‘definitely unable to return to pre-injury level’, ‘able, but not pre-injury level’, and ‘pre-injury level’. Patients’ quality of life was assessed using the 5-dimensional, 3-level EuroQol questionnaire (EQ-5D-3L), which consists of a descriptive system covering five dimensions (mobility, self-care, usual activities, pain/discomfort, and anxiety/depression), with three levels each (no problems, moderate problems, and extreme problems)^21^ ^,^ ^22^.

### Statistical analysis

Data were presented as absolute numbers with percentages (%) for dichotomous and categorical variables. Bivariate linear regression was used to assess individual factors that affect patients’ health-related quality of life. A p-value <5% was considered statistically significant.

## RESULTS

Fifteen patients (nine males and six females) diagnosed with infected pseudarthrosis of the femoral or tibial metaphysis, treated with antibiotic cement-coated plates, were eligible for the study. The mean age was 52.9 years (ranging from 24 to 72). In three (20%) cases the nonunion occurred in the proximal region of the femur, in eight (53.3%) in the distal region of the femur, and in four (26.7%), in the proximal region of the tibia. We observed hypertrophic pseudarthrosis in one (6.7%) patient, oligotrophic in 12 (80%), and atrophic in two (13.3%). On admission, ESR and CRP were elevated in all patients and malnutrition (serum albumin <3.2g/dl) was observed in five (33.3%) individuals preoperatively. All patients had or reported at least one of the following clinical or social events: smoking, diabetes, previous open or high-energy fracture, previous surgeries, poor soft tissue status, and active fistula at the site of the pseudarthrosis. The mean number of previous surgeries was 2.53 (ranging from 1 to 5). In 12 (80%) patients a plate was used in the index procedure, and in three (20%), IMN. Two (13.3%) patients had varus misalignment of the distal femur. The preoperative range of motion (ROM) ranged from 30° to 100° for the hip (range 90° to 115°) and -5° to 60° for the knee (range -15° to 90°). Table 1 presents the demographic data of the study patients.

**Table 1:** Patient demographics.

Patient	Age (years)	Sex	Comorbidities	Infected pseudo- arthrosis site	Previous implant	Number of previous surgeries
1	53	M	HIV/Poor soft tissue coverage	PT	Plate	3
2	57	M	DM / Obesity	PF	IM rod	2
3	31	M	-	PT	Double plate	2
4	29	F	-	PT	Plate	3
5	56	M	-	DF	Plate	1
6	63	F	Smoking	DF	Plate	1
7	72	M	Smoking	DF	IM rod	2
8	44	M	-	DF	Double plate	5
9	50	F	-	PF	DCS	4
10	41	M	Smoking	PF	DCS	3
11	66	M	-	PT	IM rod	2
12	48	F	Smoking	DF	Plate	3
13	68	M	Smoking	DF	DCS	5
14	39	M	Smoking	DF	DCS	1
15	24	F	-	DF	DCS	1

PT: proximal tibia; DF: distal femur; PF: proximal femur; IM: intramedullary; DCS: Dynamic Condylar Screw; HIV: human immunodeficiency virus; DM: diabetes mellitus. Source: HUA, HSL, & SOT NOVA-HMMC, 2020.

Methicillin-susceptible S. aureus was isolated in eight (53.3%) cases, E. cloacae in two (13.3%), and E. coli, Proteus mirabilis, and Streptococci viridians in one (6.7%) each. Two (13.3%) patients had negative culture and histopathology. In 12 (80%) patients, gentamicin associated with vancomycin was used together with PMMA and in three (20%), pure vancomycin. The induced membrane technique was performed in three (20%) patients, two (13.3%) with atrophic pseudarthrosis and one (6.7%) with oligotrophic. One patient (6.7%) required a local flap of the medial gastrocnemius muscle due to poor coverage conditions on the medial aspect of the affected leg.

Local infection was adequately controlled (ESR 30mm/1^st^ hour and CRP 10mg/dl) after initial treatment in 12 (80%) patients, including three (20%) treated by the Masquelet technique. Persistent drainage of the operative wound was observed in two (13.3%) patients, requiring new irrigation and debridement procedures, with adequate late resolution. In one (6.7%) patient, there was a need to add an IMN to the antibiotic coated PMMA plate to increase stability at the pseudarthrosis site. In one (6.7%) patient with proximal pseudarthrosis of the femur, the infection persisted and there was persistent drainage, despite numerous local debridement procedures, requiring removal of the implant, resection of the proximal segment of this bone, and placement of an antibiotic spacer. In general, at least one additional minor surgery, such as bone grafting and removal of loose screws, was required during the course of treatment.

**Figure 2:**
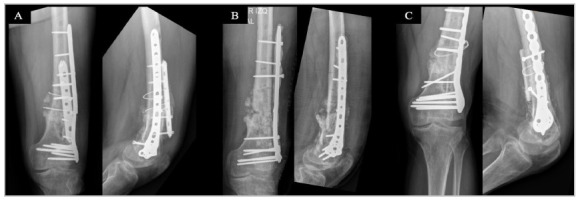
Case 2. A. Antero-posterior (AP) and lateral radiographs of the distal region of the left femur, showing osteosynthesis failure, with plaque breakage and oligotrophic infected pseudarthrosis of the distal femur metaphysis; B. The implants were removed and an antibiotic cement-coated block plate was used for stabilization. AP and lateral radiographs show persistent nonunion; C. The second reconstructive surgery was performed, with implant removal and bone healing with a structural allograft. AP and lateral radiographs of the second reconstructive surgery showing the new fixation and structural graft.

Radiographic bone healing was observed in 12 (80%) patients. In three (20%) patients, implant removal and further surgery were required. Bone consolidation ended up occurring in two (13.3%) of these patients after the implant was replaced and the induced membrane technique or structural allograft placement was performed (Figure 2). As mentioned earlier, an antibiotic spacer was used in one (6.7%) patient with a persistent infection in the proximal femoral region.

**Figure 3:**
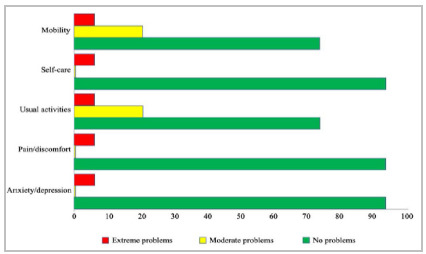
EQ-5D-3L responses of the 15 patients.

The mean postoperative follow-up time was 18 months (ranging from 8 to 37). Of the patients considered cured, none showed new symptoms of infection at the surgical site. In two (13.3%), the plate was removed after bone healing due to local pain. Reduced knee ROM (<90º) was observed in two (13.3%) patients. According to the Peek et al.^20^ modified criteria of return to pre-injury level to daily activities, 14 (93.3%) patients reported being ‘capable but not pre-injury level’ or ‘pre-injury level’. Eleven (73.3%) patients reported ‘no problems’ in all five dimensions of the EQ-5D-3L, and three (20%) patients reported ‘moderate problems’ related to mobility and usual activities. ‘Extreme problems’ in all dimensions were observed in the patient who required resection of the proximal region of the femur. The EQ-5D-3L responses are shown in Figure 3. In the bivariate analysis, persistent infection (p<0.001) was the only variable associated with reduced long-term quality of life, as measured by the EQ-5D-3L.

## DISCUSSION

This study confirms both the feasibility and the efficacy of antibiotic cement-coated plates in the treatment of pseudarthrosis of the metaphyseal region of the femur and tibia. Radiographic bone healing was achieved in 93.3% of cases, with patients reporting being close to or at the same level of functional independence as before the injury after a mean follow-up of 18 months. ‘Extreme problems’, as assessed by the application of the EQ-5D-3L score, were observed in only one patient, due to treatment failure and the need for resection of the proximal region of the femur, which required the use of a femoral spacer coated with PMMA.

Our findings are in agreement with what has been reported on the use of antibiotic cement-coated plates in the treatment of infected pseudarthrosis of the femur and tibia. Liporace et al.^23^ were the first to report the technique in a 50-year-old male patient who suffered a Vancouver B1 periprosthetic femur fracture in the immediate postoperative period of total hip arthroplasty that presented with chronic deep infection. The patient was treated through staged surgical procedures, with implant removal, reconstruction of the proximal part of the femur using a femoral spacer, fracture fixation with a locked plate coated with antibiotic cement, and, as a last stage, revision arthroplasty with a cementless nail, after eradication of the infection and clear signs of bone healing of the fracture. The patient evolved satisfactorily, with no laboratory signs of infection and no pain complaint after one year of follow-up. Other authors have presented similar and equally satisfactory results using the same technique in the management of early and late cases of IAO^10^ ^,^ ^14^ ^,^ ^24^ ^,^ ^25^.

PMMA associated with antibiotics has been widely used in the treatment of bone infections, especially in certain situations that can negatively impact the final result, such as a loose and unstable implant, highly virulent and/or multidrug-resistant organisms, and in the presence of biofilm in the maturation phase^5^ ^,^ ^7^ ^,^ ^8^ ^,^ ^11^ ^,^ ^12^. High local concentration and release of antibiotic drugs and the exothermic reaction during cement polymerization have been identified as potential benefits of using PMMA as a carrier vehicle for the elimination of local bacterial colony-forming units^24^. The ideal antibiotic for incorporation into bone cement must be selected to achieve optimal elimination of broad-spectrum bacteria. In addition, the chosen antibiotic should have adequate bactericidal activity, high specific antibacterial potency, low risks of bacterial resistance during therapy, undesirable side effects, and allergic reactions, marked water solubility, and chemical and thermal stability^11^ ^,^ ^26^. In our patients, the antibiotic cement-coated plate was prepared with gentamicin and/or vancomycin, depending on the prior identification of the pathogen. In 12 patients, the two antibiotics, which demonstrated a potential synergistic effect in vivo, were combined^27^. Aminoglycosides, especially gentamicin and tobramycin, have been shown to have adequate bacteriological and physicochemical characteristics^26^.

Infection control requires aggressive debridement of all infected tissue, which sometimes creates massive bone defects. In this situation, the two-stage induced membrane technique has demonstrated the ability to reduce dead space, add stability, and prevent resorption of the graft or bone substitute, favoring its revascularization and corticalization, with good clinical results^7^ ^,^ ^28^. The first phase of treatment should last at least six weeks before the second phase of treatment is carried out. The kinematics of release of the antibiotic used in the cement spacer is a critical aspect in this time interval. Aminoglycosides and vancomycin are the most widely used drugs, presenting a biphasic elution profile, with an initial high and rapid release, followed by a much slower, but sustained, delivery of the drug^29^ . Kelm et al.^30^ demonstrated that both gentamicin and vancomycin continue to be released from explanted spacers after three to six months of implantation. In our study, we performed the Masquelet technique in four patients, three from the beginning and one during the course of treatment, due to persistent nonunion. Bone healing and infection control occurred in all. Jia et al.^31^ reported good results in a series of reconstructions of bone defects using the Masquelet technique. Cement-coated plates with antibiotics were used as temporary stabilizers after debridement in the first stage of surgery, followed by the induced membrane technique in the second stage. Bone healing was observed in 95.9% of patients at the last outpatient evaluation. As a technical detail, these authors recommended attention to the skin when using the antibiotic cement-coated plate, especially when there is poor condition of local soft tissues, which is especially critical in the distal region of the leg.

Reconstruction of the soft tissue envelope is an important component of the treatment of long bone infected pseudarthrosis^1^ ^,^ ^7^ ^,^ ^15^ ^,^ ^31^. The goals are to ensure a tension-free suture and to establish a good local blood supply, potentially increasing the action capacity of local and systemic antibiotics^31^ ^,^ ^32^. This is especially critical in the anteromedial regions of the distal third of the leg^31^. Careful preoperative planning must be carried out to properly choose a low-profile implant, define its location, and assess the need for local or microsurgical flaps. Currently, as an adjuvant measure, negative pressure therapy (NPT) is being proposed to assist wound coverage and accelerate granulation formation, demonstrating excellent results^33^. In our study, one patient required a medial gastrocnemius muscle flap, together with fixation of the proximal tibia with an antibiotic-coated PMMA plate. We used pre-shaped anatomical locked plates in all patients and none required NPT. This was due that several factors, such as poor bone stock, osteopenia, instability due to nonunion, infection, joint stiffness, and short epiphysometaphyseal fragment that make it difficult to treat long bone metaphysis infected pseudarthrosis, hampering the acquisition of a stable environment with unblocked implants. Under these conditions, the routine use of locked plates provides sufficient local stability, improving the functionality of the construction and reducing the rate of mechanical failure^31^ ^,^ ^32^.

We recognize the existence of some limitations in the study. Notably, the study design was of a retrospective cohort, carried out in three level-I trauma centers in two different countries. Therefore, some individual factors, such as interracial variations and anthropometric characteristics, as well as small details of the surgical technique itself, may have influenced our findings. To control for these variables, we included only adult patients with infected metaphyseal pseudarthrosis of the femur or tibia with a minimum follow-up of eight months. In addition, preoperative assessment was standardized, including patient-specific factors, past medical history, physical examination, and laboratory and imaging studies. As a second limitation, there was no control group, which prevented comparisons with other treatment methods, such as bone transport or IM nails with multiple blocks^34^. However, we observed largely satisfactory results using blocked plates coated with antibiotic cement in our series, which was also observed and reported by numerous other authors^10^ ^,^ ^14^ ^,^ ^23^ ^-^ ^25^ ^,^ ^31^. It is known that the treatment of long bones infected pseudarthrosis involves distinct and well-recognized surgical steps, which include the removal of all devitalized tissue, adequate cleaning of the surgical site, obtaining a mechanically stable bone environment, regardless of the implant used, and adequate soft tissue coverage around the previous infection site^5^ ^,^ ^7^ ^,^ ^31^ ^,^ ^33^. As a third limitation, we highlight that our sample was not large, with 15 patients treated consecutively, despite the relative prevalence of IAO. However, even though this series came from three trauma centers, which deal daily with a large number of patients victimized by high-energy trauma, our study reports the largest series of cases using antibiotic cement-coated plates in the treatment of infected pseudarthrosis of the metaphysis of the femur and tibia. In any case, we prudently understand that consistent recommendations cannot be expected only from case series, so we advise cautious interpretation of our findings, as well as those of other authors who used the same technique.

## CONCLUSION

The antibiotic cement-coated plate proved to be a viable and efficient surgical technique for the treatment of infected pseudarthrosis of the femoral and tibial metaphysis.
